# Adaptation to Change Questionnaire for Nurses: Validation and New Needs in the Context of COVID-19

**DOI:** 10.3390/healthcare9121762

**Published:** 2021-12-20

**Authors:** Ana Belén Barragán Martín, María del Mar Molero Jurado, María del Carmen Pérez-Fuentes, Azucena Santillán García, Diana Jiménez-Rodríguez, Elena Fernández Martínez, Iván Herrera-Peco, África Martos Martínez, Raquel Franco Valenzuela, Inmaculada Méndez Mateo, José Jesús Gázquez Linares

**Affiliations:** 1 Department of Psychology, Faculty of Psychology, University of Almería, 04120 Almeria, Spain; abm410@ual.es (A.B.B.M.); mmj130@ual.es (M.d.M.M.J.); mpf421@ual.es (M.d.C.P.-F.); jlinares@ual.es (J.J.G.L.); 2 Department of Psychology, Faculty of Psychology, Universidad Politécnica y Artística del Paraguay, Asuncion 1628, Paraguay; 3 Department of Cardiology, Burgos University Hospital, 09006 Burgos, Spain; ebevidencia@gmail.com; 4 Department of Nursing, Physiotherapy and Medicine, University of Almería, 04120 Almeria, Spain; d.jimenez@ual.es; 5 Nursing and Physiotherapy Department, Faculty of Health Sciences, University of León, 24071 Leon, Spain; elena.fernandez@unileon.es; 6 Nursing Department, Health Sciences Collegue, Alfonso X El Sabio University, 28691 Madrid, Spain; iherrpec@uax.es; 7 Hospital Universitario Mútua de Terrassa, 08001 Barcelona, Spain; rfranco@mutuaterrassa.cat; 8 Department of Evolutionary and Educational Psychology, University of Murcia, 30100 Murcia, Spain; inmamendez@um.es; 9 Department of Psychology, Universidad Autónoma de Chile, Providencia 7500000, Chile

**Keywords:** nurses, adaptation to change, COVID-19, ADAPTA-10 questionnaire

## Abstract

Background: The worldwide pandemic caused by the SARS-CoV-2 coronavirus has challenged healthcare systems and the professionals who work in them. This challenge involves strong changes to which nurses have had to quickly adapt. Emotional and cognitive–behavioral factors influence the capacity for adaptation to change. Based on this model, the objective of this study was to validate the Adaptation to Change Questionnaire (ADAPTA-10) for identifying professionals in a population of nurses who have problems adapting to adverse situations such as those caused by COVID-19. Methods: This study was performed with a sample of 351 nurses. (3) Results: The ADAPTA-10 questionnaire was found to have good psychometric properties, and to be an effective, useful tool for nurses in research and clinical practice. The two-dimensional structure proposed in the original model was confirmed. Scales are also provided by sex for evaluation of adaptation to change; the highest scores on the emotional component were among nurses who had not personally encountered the virus. Conclusions: This instrument will be able to detect of the needs for adaptation to the new reality associated with COVID-19, as well as other situations in which nurses are immersed that demand adaptation strategies.

## 1. Introduction

The current COVID-19 pandemic caused by infection of the SARS-CoV-2 virus is a worldwide health problem. Since December 2019, the epidemic, identified first in Wuhan (China), has spread to a large number of countries [1,2], causing a huge healthcare and social emergency around the world.

Infection by SARS-CoV-2 is usually associated with respiratory disease; although, it generates a variable symptomatology of light to severe symptoms [3,4]. There are also effects on mental health that can be direct, from the disease itself, or derived from the situation the pandemic has generated, such as confinement, uncertainty, or perceived threat [5,6,7]. Among them are stress, anxiety, depression, or psychological distress [8]. Thus, there is concern for the psychological impact that COVID-19 could have, especially in vulnerable persons with pre-existing mental health affections [3,9] in those hospitalized or in difficult situations caused by isolation and the economic crisis, as well as healthcare workers [5].

There is growing concern about the mental health, psychological adjustment, and recovery of the latter in particular. These professionals have been required to work in a very complex environment, where every individual resource had to be mobilized to provide emergency care in an environment of uncertainty. Along this line, nurses have been faced with challenges escaping their control in healthcare attention during the pandemic, including insufficient staff, beds, mechanical ventilators, personal protective equipment (PPE), and others [10], which could lead to risk of exhaustion [11,12].

In this context of limited resources, nurses have had to adapt and implement innovative measures to cope with the situation. Strategies would be directed at optimizing the use of PPE, reducing spread of the disease and generating a safe care environment [13]. It is important to highlight the role of nurses in preventing COVID-19 and attention of infected patients.

In previous studies in China, first-line healthcare professionals showed high risk of developing unfavorable mental health and need of psychological support or specifically designed intervention [14,15]. In fact, higher levels of psychiatric symptoms associated with the pandemic have been found among health workers than in the general population, so periodic evaluation of the mental health of these workers is recommended [16], as well as development of measures and interventions designed to assist in their psychological wellbeing during and after the pandemic [15].

It was known in occupational health that priority preparation was necessary to cope with a pandemic; however, it seems that this crisis has exceeded expectations. Psychosocial interventions must extend beyond the acute period of the crisis, as it is likely that traumatic stress and emotional problems of healthcare professionals will become more frequent in the future [17]. Similarly, promotion of personal resilience resources (resistance, optimism, and emotional competence) is useful in promoting the psychological health and wellbeing of professionals, as well as making organizations more resistant [18].

### 1.1. Adaptation to Change

Study of those subjected to adverse situations has repeatedly demonstrated that while some individuals show strong difficulties in adapting, others, even under the most difficult circumstances, are able to maintain an adequate level of functioning and wellbeing [19,20].

In such adverse situations, it is important to keep in mind the concept of adaptation with regard to functional change in response to environmental stimuli, where positive adaptation to adversity is not a completely innate trait, and therefore, can be learned and developed with active reformulation of life’s challenges [21]. Thus, adversity can be transformed into opportunity from a perspective of committed action, practical wisdom, individual courage, resilience, and capacity for adaptation [22]. Therefore, individual reaction patterns must be known to learn the most effective coping mechanisms and protect the subject from psychological alteration [23].

Psychological adaptation of nurses has been analyzed in natural disasters, as a high percentage of these professionals usually develops psychological alterations, such as posttraumatic stress syndrome, after working under such circumstances. It is known that nurses who have suffered from posttraumatic stress show changes in emotions, as well as coping skills, social relations, and interaction skills, in addition to increased psychological burden [24]. It seems that women adapt worse to highly stressful events [25].

### 1.2. Factors in Adapting to Change

Everyone must adapt more or less to the changing environment every day. According to the model by Pérez-Fuentes et al. [7], adaptation to change to environmental demands requires an emotional state contrary to suffering and distress, and the ability to manage and undertake actions that promote adjustment to new challenges. Thus, two factors are involved in one’s ability to adapt. The first is cognitive–behavioral and refers to competence in managing and taking action in an appropriate response to challenging everyday situations. The second is the emotional dimension linked to feelings such as anxiety and depressive symptoms and tolerance to uncertainty while adapting. The capacity for adaptation to change includes responses of both types. This model makes it possible to deepen knowledge of the capacity to adapt to uncertain everyday situations in a changing environment and its repercussions.

Emotionally, coping with multiple challenges can cause high levels of anxiety, stress and secondary posttraumatic stress syndrome in nurses [26,27]. Secondary posttraumatic stress syndrome refers to emotions and behaviors that appear as the result of having echoed a traumatic event experienced by another person [28], so a highly stressful situation can affect more than just the one who suffers directly from it [29]. Thus, nurses, immerged in a job context with a strong emotional and affective content, chronically cope with their patients’ stress, which can affect the workers themselves if they are unable to cope with the change as a positive challenge [30]. In addition, as part of inadequate adaptation, depressive symptoms may appear, threatening healthcare professionals’ health and wellbeing. These symptoms are due to contextual and stress factors, so the work environment and nursing care are closely related to their appearance, especially when carried out in uncertain [31] and poorly compensated [32] situations.

In adaptation, tolerance to uncertainty is connected to anxiety and stress in highly demanding situations [33]. This variable refers to how people understand and process information in uncertain situations and how they respond to it with cognitive–behavioral and emotional reactions [34]. The way in which people perceive and cope with uncertainty becomes relevant to their ability to adapt [35]. Applied to clinical settings, uncertainty is inherent to healthcare practice. In the scope of nursing management, uncertainty is described as perceived environmental uncertainty in the context of rapidly changing, turbulent practice environments [36]. However, as nurses act on the basis of their decisions, and the decisions have implications for patient health, it is important to understand nurses’ uncertainty in their daily practice [37]. It is known that strategies for managing uncertainty in both individual and social contexts include proactive searches for solutions, collaborating, and seeking evidence [38], as well as the use of motivational strategies to improve the work alliance [39]. Thus, coping focused on the problem has been shown to be relevant to ensuring proper functioning of nurses confronted with highly stressful or traumatic situations, favoring their adaptation and proper performance of their daily activity [24]. Emotional management also enables the effect of stress to be buffered and coped with, which makes this variable fundamental, both in the general population [40] and in healthcare professionals, to adequately manage new situations brought on by the pandemic [41,42]. Finally, in environments and scenarios that suddenly change, it becomes necessary to remain highly aware of elements in them within a space and a time, understanding their meaning and projecting their state to a near future [43]. This is especially true in healthcare, where there may be highly disruptive work flows impeding full attention to the situation, and which therefore, affect worker cognition [44].

It is as necessary to have evaluation tools for detection and intervention in psychological crises derived from stress, anxiety, depression, and exhaustion of healthcare workers as it is to have tools for the prevention of transmission of diseases [45]. Nurses are a labor force that is hard to replace, and the deterioration of their health can lead not only to decrease in their quality of life, but could also affect the quality of care they give the population [46].

### 1.3. Objective

The COVID-19 pandemic has challenged healthcare systems, and this can compromise the psychological wellbeing of healthcare professionals [15] by generating new demands and scenarios both in the workplace and outside of it. The need to explore factors related to better adaptation of nurses emerges from this. Thus, the objective of this study was to validate the ADAPTA-10 adaptation to change scale for these professionals.

## 2. Materials and Methods

### 2.1. Participants

The sample was comprised of a total of 357 currently employed Spanish nurses. Implementation of the questionnaire included control questions for detecting random or incongruent answers, which eliminated six subjects, so that the final sample was made up of 351 nurses. The mean age of the sample was 40.91 (*DT* = 11.02), in a range of 21 to 67; 86% (*n* = 302) of them were women and 14% (*n* = 49) men, whose mean ages were 40.81 (*DT* = 11.21) and 41.49 years old (*DT* = 9.89), respectively.

### 2.2. Instruments

An ad hoc questionnaire was prepared to collect the sociodemographic data, including items on age, sex, marital status, etc. Questions related to their personal experience with COVID-19 were also asked to find out whether they had been diagnosed with COVID-19, whether there had been anyone in their immediate surroundings infected by SARS-CoV-2 or whether the pandemic had caused them any personal economic impact.

The Spanish adaptation of the General Health Questionnaire-28 (GHQ-28) [47,48] was applied. This scale, divided into four subscales, consists of 28 items, seven on each of the subscales, which provides a score in somatic symptoms (Subscale A), anxiety and insomnia (Subscale B), social dysfunction (Subscale C), and severe depression (Subscale D). Each one of the items has four possible answers, where the participants must mark their answer based on their situation in recent weeks. The instrument’s reliability was found to be ω = 0.93, for the complete scale, and each of the subscales: somatic symptoms (ω = 0.86), anxiety and insomnia (ω = 0.90), social dysfunction (ω = 0.81), and depression (ω = 0.91).

The questionnaire designed to measure the individual’s ability to adapt to change in novel situations was called the Adaptation to Change Questionnaire (ADAPTA-10) [7]. This instrument consists of 10 items divided into two factors. The first is related to anxiety and distress which can appear when changes occur (emotional factor) and the other to the ability to control, manage, and act in different situations (cognitive–behavioral factor). The answers were rated on a five-point Likert-type scale (from “not at all” to “very much”).

### 2.3. Procedure

Data in this cross-sectional study were found by a snowball design. The questionnaire was implemented on an online platform as a CAWI (Computer Aided Web Interviewing) interview, which facilitated its diffusion on social networks and instant messaging among groups of nurses. Data were collected in the seventh and eighth weeks of confinement in Spain, that is, from 1 to 12 May 2020.

### 2.4. Data Analysis

Data were analyzed by studying the structure on the theoretical basis of the *Escala de Adaptación al Cambio* [7] with exploratory and confirmatory factor analyses of the theoretical Adaptation to Change model proposed. The Confirmatory Factor Analysis (CFA) for the original model took the following fit indices as measures: *χ*^2^/*df*, Comparative Fit Index (CFI), Tucker–Lewis index (TLI), and Root Mean Square Error of Approximation (RMSEA) with a 90% Confidence Interval (CI). The χ^2^/*df* index was used considering below five acceptable [49], CFI and IFI over or near 0.90, and for the RMSEA below or very near 0.08 [50]. As a general rule, good fit of the model would be when the 2/*df* ratio ≤ 3; TLI > 0.90, CFI > 0.95, RMSEA ≤ 0.05. The various models proposed in the original study were analyzed using the Akaike Information Criteria [51] for model selection.

Reliability of the new scale was analyzed using Cronbach’s Alpha [52], the Spearman–Brown coefficient, and the intraclass correlation coefficient.

Finally, the invariant factor structure proposed across gender (men/women) was analyzed. First goodness of fit of the structures was tested separately in the two subsamples (Models M0a—Men and Model M0b—Women). The four nested models resulting were evaluated: (a) Model 1. Both subsamples were considered simultaneously with free estimation of parameters. (b) Model 2. Metric invariance was show. (c) Model 3. Scalar invariance was shown. (d) Model 4. Strict invariance. Without a consensus criterion for determining the criteria to be used to evaluate the difference in fit between the different nested models [53]. For evaluation of fit, this study used the ΔCFI. The model is thus interpreted as completely invariant if the value found in the ΔCFI is below 0.01 [54].

Similarly, the validity of the construct was analyzed by correlating the items and factors with other instruments that measure related aspects.

The analyses were performed with the SPSS statistical package version 23.0 for Windows and the AMOS 22 program.

In addition, a two-stage cluster analysis was carried out for case classification, and identification of possible COVID-19 scenarios. The number of clusters was estimated by automatic classification. This procedure automatically determines the optimum number of clusters, using the criterion specified for clustering, in this case, log-likelihood.

After the cases had been classified, an ANOVA was performed to find out whether there were any differences between the clusters identified with respect to the adaptation to change component (emotional and cognitive–behavioral).

### 2.5. Ethical Approval and Informed Consent

This study was evaluated and approved by the University of Almería Research Ethics Committee (Ref. Ref. UALBIO2020/032). The participants acted voluntarily, giving their express consent by marking a box provided for it on the screen. Before participation, they were informed of the objectives of the study and the confidentiality and anonymity of their answers as per current Spanish legislation on protection of personal information.

## 3. Results

### 3.1. Preliminary Analyses and Exploratory Factor Analysis of ADAPTA-10

First, descriptive statistics were analyzed for the items in the original ADAPTA-10 Model, which were within the limits of normality and, therefore, have a normal distribution. According to the criteria of Finney and DiStefano (2006), the maximum permissible values for asymmetry and kurtosis are 2 and 7, and in this case, they were −1.02 and 2.63, respectively (Table 1).

Coinciding with the original model, the Principal Components Analysis revealed the existence of two components with eigenvalues over 1, as observed in the scree plot (Scree Test), which showed the presence of two factors with 3.8 and 2.11, followed by a third value below 1 with 0.84. Thus, Table 2 shows two components, which correspond to the Emotional Component and the Cognitive–Structural Component in the original model, with five items each, all with weights over 0.65, and explaining 59.11% of the variance (Table 1).

Reliability of the model was analyzed using the Spearman–Brown Coefficient *p* = 0.50 and the Cronbach’s alpha, where α = 0.81 for the total scale. For analysis of temporal stability, the intraclass correlation coefficient (ICC) and its confidence interval (CI), were analyzed, where results for adaptation to change were 0.81 (CI = 0.78–0.84).

### 3.2. Confirmatory Factor Analysis of the Original ADAPTA-10 Model

**Figure 1:**
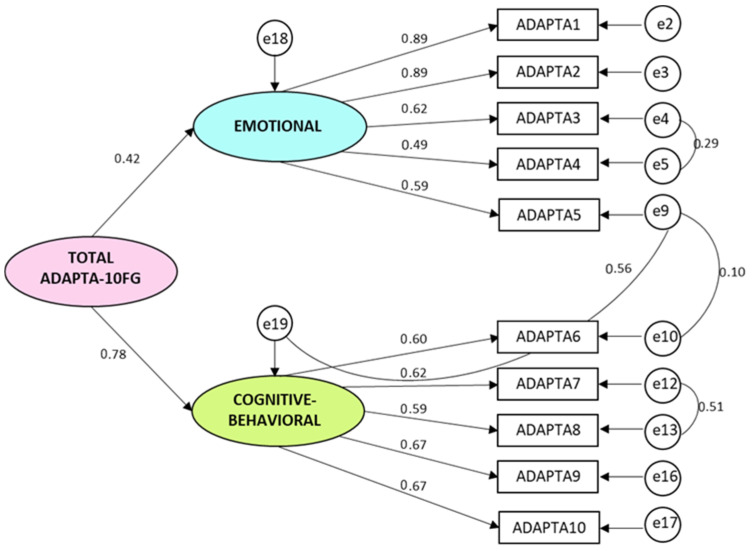
Confirmatory Factor Analysis ADAPTA-10FG Model.

The two-factor model with a general adaptation factor and 10 items (Figure 1), shows very adequate values: *χ*^2^ = 64.89; *df* = 30; *χ*^2^/*df* = 2.16; Comparative Fit Index CFI = 0.97; Tucker–Lewis Index TLI = 0.96; Root Mean Square Error of Approximation RMSEA (Confidence Interval CI 90% Inf.-Sup.) = 0.058 (0.038–0.077). Thus, the Adapta-10 Model shows adequate fit, and the difference between the AIC Default model = 114.896 and the AIC Saturated model = 110.000 is also very small, showing that this is probably the best model according to the Akaike model selection criteria.

Table 2 shows the values for the six different models in the analysis of variance across sex. In all cases, the ΔCFI is below 0.01, so configural, metric and strict invariance may be accepted, except in the last model, where strong invariance does not meet the criteria for its acceptance.

Part of the validity of the construct was examined through convergent validity relating health problems with the GHQ-28 and the capacity for adapting with the ADAPTA-10. The GHQ-28 was selected for convergent validity because the Adaptation to Change Scale includes the emotional and cognitive–behavioral dimensions, and individuals who have a poor capacity for adaptation to change do not generate responses adjusted to environmental stressors. Therefore, they end up developing psychological symptoms and alterations, and vice versa, individuals who have psychological health problems could cope differently with change, since their interpretation of situations or of changing contexts could be distorted, and this in turn, would hinder their adaptation.

The correlations with construct validity analyzed back the validity of the ADAPTA-10 questionnaire in all cases, showing that direct scores on the GHQ-28 and ADAPTA-10 questionnaires are significantly (*p* < 0.01) and negatively related.

Table 3 gives scales for evaluating adaptation to change in the Spanish nursing population, and also by sex.

### 3.3. Adaptation to Change in Nursing in a COVID-19 Context

**Figure 2:**
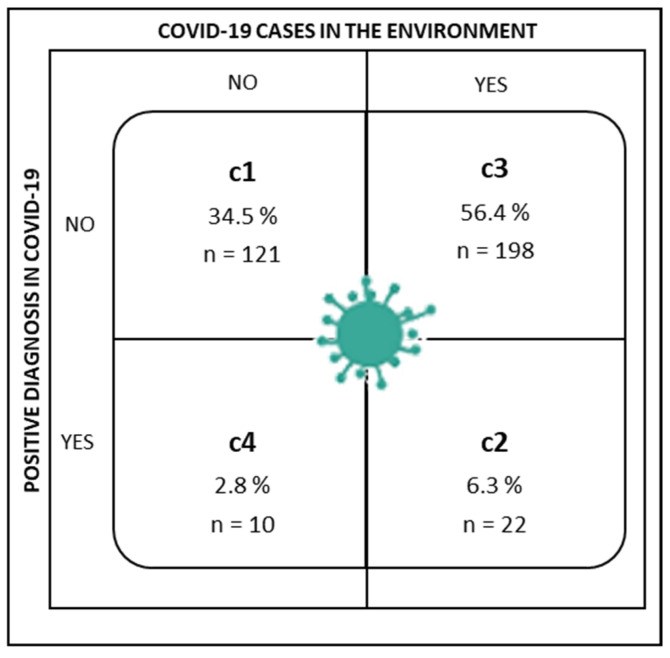
Distribution of the sample by COVID-19 scenarios.

Participants were asked whether they had been diagnosed with COVID-19 (Have you been diagnosed with COVID-19?), and whether anyone close to them had COVID (Does or did anyone close to you have COVID-19?; referring to the existence of infection in the individual’s close social setting, family, friends, etc.). The answers were yes/no in both cases. Thus, the cluster analysis identified the following groups by possible combinations of answers to these two questions on the COVID-19 context (Figure 2).

Cluster 1 (C1) was made up of nurses who said they had not been diagnosed with COVID-19, nor did anyone close to them have a positive diagnosis.

Cluster 2 (C2) grouped nurses who answered both questions affirmatively; they had been diagnosed with COVID-19 and there were cases close to them.

Cluster 3 (C3) assembled nurses who had not been diagnosed with COVID-19, but did have someone close to them who had been.

Finally, Cluster 4 (C4) included nurses who had been diagnosed with COVID-19, but no one close to them had been.

**Figure 3:**
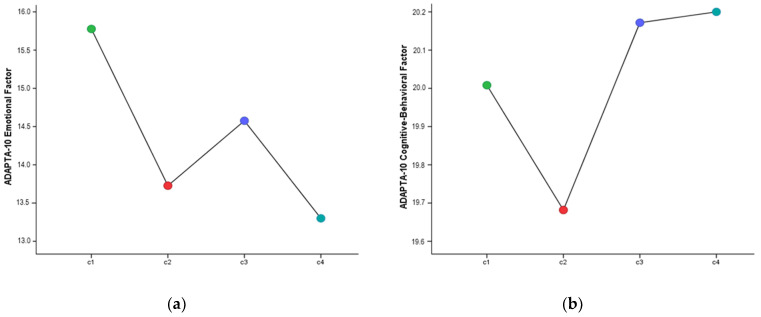
Adaptation to change by COVID-19 clusters. [Note: (**a**) Emotional Factor, (**b**) Cognitive–Behavioral Factor].

The results of the comparison of means performed to check for differences between the clusters, both in the emotional and cognitive–behavioral factors of adaptation to change, are reported below. As shown in Table 4 and Figure 3, there were significant differences between the groups for the emotional factors of adaptation to change (*F*_(3,347)_ = 3.25, *p* < 0.05, *η*^2^*_p_* = 0.027). The specific differences are in favor of C1 compared to C2 and C3, where the first had a significantly higher mean score for the emotional factor of adaptation to change. However, the cognitive–behavioral factor did not show any differences in the various COVID-19 situations.

## 4. Discussion

The objective of this study was to validate the ADAPTA-10 Questionnaire for evaluating the capacity for change in a population of nurses. The psychometric properties of the instrument showed it to be adequate.

Following the factor structure of the original questionnaire (ADAPTA-10) [8], validation for the nursing population was made up of two factors. The first of them, the emotional factor, contained items referring to anxiety, depression, and tolerance to uncertainty. Dealing with new demands arising daily generates anxiety and stress, to which stress experienced by other persons is added [26,29]. The COVID-19 pandemic has generated a critical situation for the healthcare sector, which has had to face not only their own anxiety, but also that of patients and coworkers in a general climate of uncertainty [5,6,7,8]. Added to this is daily exposure to the virus, with the consequent worry about their own health and fear of carrying the infection to family and/or patients, longer hours of work, stress, burnout, and emotional suffering, as well as the frequent depressive symptoms in highly stressful, unknown contexts [31,55], which can impede adaptation. Therefore, tolerance to uncertainty, which has been shown to be a variable included in both factors of the questionnaire structure, is established as relevant to emotional and cognitive–behavioral response favoring adaptation [34,35]. The questionnaire’s cognitive–behavioral factor, along with tolerance to uncertainty, concerned attention, coping, and emotional management made up the aspects evaluated. This assumes that being aware of changes, especially in situations with high workflow such as healthcare [44], along with the ability to cope with emotions that emerge during adaptation and select responses that face the problem directly [24] make it possible to detect and adapt to new demands that appear daily. Occupational risk prevention services can have an important role in the prevention of psychosocial risks in the workplace, and nurse attention programs are a relevant resource, if there is disposition to do so and they are invested in.

Lastly, the differences in the dimensions of the questionnaire on nurses’ adaptation to change by COVID-19 diagnosis, both personal and of someone close to them, were analyzed. Nurses in the first group, made up of those who had not had COVID-19 nor had anyone close to them, were found to have significantly higher scores on the emotional factor than those who had someone close to them diagnosed with COVID-19, or compared to nurses who had had the virus and also someone close to them. This suggests that more negative feelings arise in the adaptation process of nurses who have not personally confronted the virus. That is, there was a more emotionally dysfunctional response to the nervousness caused by uncertain situations among those who for the time being have not had to cope with the disease.

Lastly, a series of limitations of this study should be mentions, such as the fact that it was a cross-sectional study and was limited to a Spanish population. In spite of its limitations, we consider that it would be of interest to perform future studies that would make the tool available in occupational risk prevention servers so that the detection of certain risk symptoms, such as anxiety or stress from maladaptation to a situation that has occurred. It would even be of interest if it could be administered in other countries for comparison. Administration of the instrument to other healthcare personnel, such as doctors, physiotherapists, psychologists, could also be considered. Finally, the sample distribution should be mentioned. In this case, only 14% of the participants were men. However, this is a characteristic of nursing in Spain, in which most of the nurses are women. This instrument is not specific to COVID-19, but it was a contextualized tool. Therefore, it should be reanalyzed in the future, when the special situation of the health emergency has ended.

## 5. Conclusions

The worldwide situation in the COVID-19 pandemic has made it necessary to develop and validate scales to analyze anxiety or stress, for example, under these conditions. Scientific evidence has further demonstrated the need for scales that can determine as well and as fast as possible how the population is managing psychologically under these conditions. Nurses have performed an indispensable role in caring for infected patients, in some cases very severe and requiring hospitalization, and above all offering attention to community outpatients as well as the general public.

Therefore, such situations of emotional and psychological impact as they have been exposed to, require the development of instruments that can detect anxiety or depressive emotional symptomatology along with the cognitive–behavioral factors (tolerance to uncertainty, attention, coping and emotional management). It is fundamental to prepare healthcare personnel with training and information to detect whether situations, such as those generated by the COVID-19, can be causing emotional or cognitive–behavioral repercussions. This would enable prevention programs to be designed for emotional management appropriate to situations of uncertainty and even adequate cognitive strategies for managing stress or anxiety.

In conclusion, this study has important implications, as it presents a tool that can be available for nurses in their various services. It is a scale which enjoys good psychometric properties, valid and reliable, and can provide useful information on the state of health of nurses and their capacity for adaptation and adjustment to demands. We therefore encourage other authors to adapt and validate this instrument in other languages in benefit of the mental and physical health of nurses, and so prevent repercussions in their place of work which can lead to more severe situations, such as burnout, and help keep intact a work force as difficult to replace as nurses.

The validation of the Adaptation to Change Questionnaire, Adapta-10 showed that adaptation to change to new daily demands follows the original model structure, with one emotional and another cognitive–behavioral component. Therefore, this tool enables the needs for adaptation of nurses to be identified, and thereby detect whether situations like the one generated by COVID-19 can be causing emotional or cognitive–behavioral repercussions in nurses. Furthermore, beyond COVID-19, every individual must adapt to a more or less highly changing environment. The lack of capacity for recovery of one’s previous state of wellbeing before the transcendental life circumstances has been shown to have long-term psychological effects. This scale can deepen knowledge about this capacity and its repercussions on the uncertainty of everyday situations, not necessarily linked to the pandemic. It is also valid for establishing the level of this variable in nurses, enabling intervention programs to be developed to strengthen their capacity for adaptation, and therefore, promote better adjustment to social and work demands. The scale could be used for prevention and early diagnosis of nurses’ mental health problems derived from poor adaptation to adverse situations similar to those triggered by the COVID-19 pandemic.

## Figures and Tables

**Table 1:** Descriptive statistics. Factor structure, communalities (*h*^2^) eigenvalues, Cronbach’s alpha and percentage variance explained. Extraction method: Principal components factoring (Varimax rotation).

Items	*n*	*M*	*SD*	Skewness		Kurtosis		F1	F2	*h* ^2^
				Statistics	Std. Error	Statistics	Std. Error			
Adapta 1	351	2.66	1.04	0.22	0.13	−0.55	0.26	0.87		0.76
Adapta 2	351	2.59	1.07	0.37	0.13	−0.47	0.26	0.88		0.77
Adapta 3	351	3.08	1.19	−0.08	0.13	−0.86	0.26	0.75		0.57
Adapta 4	351	3.44	1.11	−0.24	0.13	−0.76	0.26	0.67		0.45
Adapta 5	351	3.12	1.05	−0.10	0.13	−0.65	0.26	0.66	0.43	0.51
Adapta 6	351	3.66	0.83	−0.44	0.13	0.17	0.26		0.69	0.48
Adapta 7	351	4.42	0.61	−1.02	0.13	2.63	0.26		0.82	0.68
Adapta 8	351	4.42	0.61	−0.94	0.13	2.18	0.26		0.80	0.66
Adapta 9	351	3.97	0.76	−0.81	0.13	1.48	0.26		0.71	0.51
Adapta 10	351	3.61	0.86	−0.41	0.13	0.02	0.26	0.45	0.65	0.51
Percentage variance explained								38.02%	21.09%	
Kaiser–Meyer–Olkin									0.81	
Bartlett’s sphericity								*χ*^2^ (45) = 1379.37, *p* < 0.000		
Cronbach’s Alpha								0.83	0.78	0.81

**Table 2:** Multigroup analysis of invariance across sex (men/women).

Model	*χ* ^2^	gl	*χ*^2^/gl	Δ*χ*^2^	CFI	ΔCFI	IFI	RMSEA (CI 90%)
M0a (men)	106.597 (*p* = 0.000)	60	1.776		0.966		0.967	0.047 (0.032–0.062)
M0b (women)	106.597 (*p* = 0.000)	60	1.776		0.966		0.967	0.047 (0.032–0.062)
M1 (base model set)	111.491 (*p* = 0.001)	68	1.639	0.137	0.969	0.003	0.969	0.043 (0.028–0.057)
M2 (SF)	113.455 (*p* = 0.001)	69	1.644	−0.005	0.968	0.001	0.968	0.043 (0.028–0.057)
M3 (SF + Int)	117.366 (*p* = 0.000)	71	1.653	−0.009	0.967	0.001	0.966	0.043 (0.029–0.057)
M4 (SF + Int + Err)	167.155 (*p* = 0.000)	85	1.966	−0.313	0.941	0.026	0.941	0.053 (0.041–0.064)

Note: FS = Factor Saturation, Int = Intercepts, Err = Errors.

**Table 3:** Scales for the nursing population: general and by sex.

		General	Men	Women
-	*M*	34.98	35.84	34.85
	*DT*	5.72	6.32	5.61
	Min	19	19	20
	Max.	50	48	50
Percentiles	10	28	27	28
	20	30.4	30	30.6
	30	32	33	32
	40	34	35	34
	50	35	37	35
	60	37	38	36
	70	38	40	38
	80	39	41	39
	90	42	43	42
	95	45	46	44.85
	99	48.5	-	48.94

**Table 4:** Adaptation to change, by COVID-19 cluster. Descriptive statistics.

ADAPTA-10		c1	c2	c3	c4	*F*	*p*	*η* ^2^ *_p_*
Emotional Factor	*M*	15.78	13.73	14.58	13.30	3.25	0.022	0.027
	*SD*	4.39	4.47	4.02	3.59			
Cognitive–Behavioral Factor	*M*	20.01	19.68	20.17	20.20	0.27	0.849	0.002
	*SD*	2.66	2.16	2.82	2.09			

## Data Availability

The data that support the findings of this study are available from the corresponding author upon reasonable request.
